# Natural Polymer Chitosan as Super Disintegrant in Fast Orally Disintegrating Meloxicam Tablets: Formulation and Evaluation

**DOI:** 10.3390/pharmaceutics13060879

**Published:** 2021-06-15

**Authors:** Gailute Draksiene, Brigita Venclovaite, Lauryna Pudziuvelyte, Liudas Ivanauskas, Mindaugas Marksa, Jurga Bernatoniene

**Affiliations:** 1 Department of Drug Technology and Social Pharmacy, Medical Academy, Lithuanian University of Health Sciences, Sukileliu pr. 13, LT-50161 Kaunas, Lithuania; gailute.draksiene@lsmuni.lt; 2 Department of Clinical Pharmacy, Medical Academy, Lithuanian University of Health Sciences, Sukileliu pr. 13, LT-50161 Kaunas, Lithuania; brigita.venclovaite@lsmuni.lt; 3 Institute of Pharmaceutical Technologies, Medical Academy, Lithuanian University of Health Sciences, Sukileliu pr. 13, LT-50161 Kaunas, Lithuania; lauryna.pudziuvelyte@lsmuni.lt; 4 Department of Analytical and Toxicological Chemistry, Lithuanian University of Health Sciences, LT-50161 Kaunas, Lithuania; liudas.ivanauskas@lsmuni.lt (L.I.); mindaugas.marksa@fc.lsmuni.lt (M.M.)

**Keywords:** tablets, chitosan, meloxicam, disintegrating, polymer, stability studies, solubility

## Abstract

The aim of the present investigation was to formulate fast disintegrating tablets of meloxicam by wet granulation technique using medium molecular weight chitosan. The orally disintegrating tablets of meloxicam with chitosan showed good mechanical and disintegration properties and good dissolution rate when prepared in tablet press using 10.8 kN and 11.0 kN compression force. Chitosan is a suitable biopolymer to moderate the disintegration process in orally disintegrating tablets.

## 1. Introduction

A fast-disintegrating tablet is a solid dosage form that disintegrates in the mouth without water in less than 1 min and leaves a pleasant sensation in the mouth [1,2]. When such a tablet is put in the mouth, saliva easily gets into the pores and quickly dissolves it. A small volume of saliva is sufficient to dissolve the tablet in the mouth cavity and water is not needed [3]. Orally disintegrating tablets are a convenient form of usage for pediatric and geriatric patients, for mentally ill, uncooperative patients, and for traveling patients who do not have immediate access to water [4]. Tablets of this type can be used to improve bioavailability of poorly soluble substances. Some of the drug is absorbed in the mouth, pharynx, and esophagus as the saliva passes down into the stomach; the bioavailability of the drug is significantly greater than those observed in conventional tablet dosage forms [5]. Any pre-gastric absorption avoids first pass hepatic metabolism and increases the bioavailability of the drug [2].

**Figure 1:**
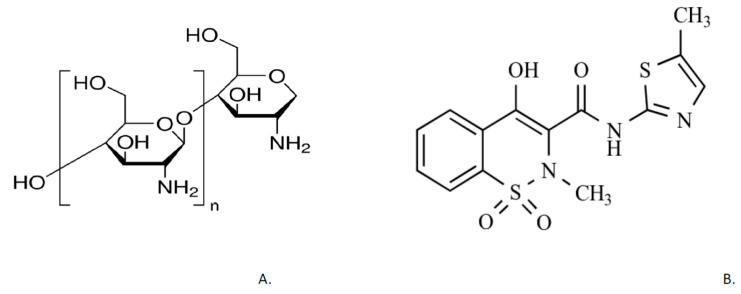
Chemical structure of chitosan (**A**) and meloxicam (**B**) [15,16]. CC BY 4.0 license.

To achieve faster disintegration, the tablet manufacturers use disintegrants, which break the tablet matrix into smaller fragments in the presence of saliva [1,6,7,8]. The disintegrants can be synthetic and natural. Advantages of natural substances over synthetic are numerous: local availability from a renewable source, low cost, biodegradable, and eco-friendly [6,8,9]. Medium molecular weight chitosan is one such substance (chemical structure represented in Figure 1A). Chitosan is a linear binary heteropolysaccharide formed of β-1,4-linked glucosamine with various degrees of N-deacetylation, obtained by *N*-deacetylation of chitin. Chitin is naturally derived from crab and shrimp shells and mushroom cell walls [10]. The molecular weight and degree of deacetylation are the key parameters that affect solubility, viscosity, coagulation, and heavy metal ion chelation of chitosan [11]. Chitosan is a unique cationic polysaccharide, well-known for its antioxidant, antimicrobial, lipid-lowering activity, film-forming, and gelling properties [12]. It can be utilized to serve several functions in pharmaceutical formulations: as a binder in wet granulation, a diluent in direct compression, a tablet disintegrant, and as a permeation enhancer. Using *N*-trimethyl chitosan with high or medium molecular weight to form hydrogels showed great success. Chitosan hydrogels display good water—holding capacity, great rheological characteristics, and strong adherence to the mucosal membrane. Sol-gel transition occurred at 32.5 °C within 7 min [11]. As chitin is a slowly biodegrading and poorly soluble substance, by deacetylation a more soluble polymer is obtained, which is suitable for production of fast disintegrating tablets [13]. The research has demonstrated the advantage of Callinectes chitosan as disintegrant over corn starch [14].

As an active ingredient for tablet manufacturing, meloxicam was chosen. Meloxicam (chemical structure represented in Figure 1B) is a substance that belongs to the group of non-steroidal anti-inflammatory drugs, oxicams. It has been shown, especially at its low therapeutic dose, to selectively inhibit COX-2 over COX-1 [17]. Meloxicam has analgesic, antipyretic, and anti-inflammatory properties and is used for the symptomatic treatment of the musculoskeletal system diseases. The dose used to treat acute pain is 7.5–15 mg. Recently, meloxicam has been considered as a potential drug for the prevention and treatment of colorectal polyps and/or cancer. It is one of the few NSAIDs approved for use in animals [18]. Meloxicam is a suitable active ingredient in the form of orally disintegrating tablets when attempting to achieve the proper absorption in the treatment of acute pain. Meloxicam is practically insoluble in water and biological fluids. The low solubility results in poor bioavailability following oral administration [19]. Meloxicam has a better solubility at higher pH, which is characteristic of the oral mucosa and small intestine. The bioavailability of meloxicam may be enhanced by the absorption of the drug in the oral cavity and also by pregastric absorption of saliva containing the dispersed drug that passes down into the stomach [17].

The disintegration time of tablets is influenced not only by the use of the disintegrant but also by manufacture methods and the compression force applied in the tablet press. To reduce the hardness and mechanical strength of the tablet a lower compression force is often used in the tablet press. Lower tablet hardness as well as higher tablet porosity can shorten tablet shelf life. Excessive compression may destroy the disintegrating effect and the porous structure of the tablets. To combine the rapid disintegration and mechanical strength of fast disintegrating tablets it is necessary to apply an appropriate tablet press compression force [2,20]. Fast disintegrating tablets can be manufactured by various methods, but the simplest are direct compression and wet granulation. The direct compression method is simple and economical; however, it can only be used for powder mixtures with good technological characteristics, and the produced tablets are compact, without porous structure, which affects the rate of disintegration and release of the active substance [2,21]. Wet granulation improves the flowability and pouring of the tableting mixture. The wet granulation tablet has a porous structure, which is crucial for the rapid disintegration of the tablets. Orally disintegrating tablets of meloxicam were selected for the study due to its low therapeutic dose and the solubility characteristics. The tablets were prepared using the intra/extra wet granulation method and different compression forces in a tablet press.

We chose the medium molecular weight chitosan for our studies due to its medium viscosity, good solubility in aqueous medium, and suitable rheological and mucoadhesive properties. As Kouchak M and authors presented medium molecular weight chitosan showed good viscosity: medium molecular weight chitosan with degree of deacetylation 92%, viscosity of 1% solution in 1% acetic acid = 715 cP compared with low molecular weight chitosan with degree of deacetylation 98%, viscosity of 1% solution in 1% acetic acid = 22 cP and high molecular weight chitosan with 96% degree of deacetylation, viscosity of 1% solution in 1% acetic acid = 1234 cP [22].

To compare results and substantiate non-ionized form of chitosan as a disintegrant in fast orally disintegrating tablets widely recognised disintegrants such as croscarmellose sodium and sodium starch glycolate were used. Croscarmellose sodium is partly O-(-carboxymethylated) cellulose. Sodium starch glycolate is chemically modified starch [23]. We chose non-ionized form of medium molecular weight chitosan because of its stability. We also wanted to avoid unpleasant smell of tablets, provided by acetic acid, when chitosan is dissolved in acetic acid solution in order to make ionized form of chitosan. Goel H. et al. examined the influence of ionized chitosan in acetic acid and glycine complex for disintegration time of fast orally disintegrating tablets and compared the results with croscarmellose sodium and sodium starch glycolate. Since glycine itself can be used as a disintegrant in orally disintegrating tablets [24,25,26], Goel H. et al. evaluated disintegrating properties of two disintegrants combined. Researchers proved that orally disintegrating tablets made with ionized chitosan and glycine complex using higher crushing strength display lower disintegration time compared to croscarmellose sodium and sodium starch glycolate [27].

Changes of technological properties are easier to make than changing the composition of granules in the technological process, so our object was to prove that technological characteristics can be used to drastically change properties of tablets, without changing their composition. We detected the limit of crushing strength in the tablet press, that allows to form good quality fast orally disintegrating tablets using only medium molecular weight chitosan as a disintegrant in order to reconcile fast disintegration and proper mechanical strength of tablets. Prepared tablets were evaluated for post-compressional parameters like drug content, weight variation, compact density, crushing strength, friability, wetting time, water absorption, disintegration time, and in vitro drug release.

## 2. Materials and Methods

### 2.1. Materials

Medium molecular weight chitosan (Sigma Aldrich, Steinheim, Germany); Meloxicam (Iroko Pharms LLC, Tvinbrook, MD, USA); magnesium stearate (AppliChem, Darmstadt, Germany); mannitol (AppliChem); sorbitol (AppliChem); microcrystalline cellulose (Sigma Aldrich); sodium dihydrogen phosphate dihydrate (Sigma Aldrich); disodium hydrogen phosphate (Sigma Aldrich); methanol (Bárta a Chilar, Roznov pod Radhostem, Czech), croscarmellose sodium (Sigma Aldrich), sodium starch glycolate (Sigma Aldrich).

### 2.2. Formulation of Fast Disintegrating Tablets of Meloxicam

Fast disintegrating tablets were prepared by wet granulation technique using medium molecular weight chitosan, croscarmellose sodium, and sodium starch glycolate. The list of ingredients is given in Table 1.

The active pharmaceutical ingredient meloxicam was mixed with the intragranular disintegrant (formulation F2, F3, F4) or without it (formulation F1) and with the diluents microcrystalline cellulose, intragranular sorbitol, and mannitol. All the components were blended and allowed to pass through 40 mesh. To this mixture ethanol was added, the wet mass passed through 30 mesh and dried at 45 °C. The dried granules were again passed through 30 mesh. The dried granules were mixed with extragranular excipients (disintegrant, diluent, and lubricants). The granules were compressed at a pressure of 10.6 kN; 10.8 kN; 11.0 kN; 11.2 kN; 11.4 kN; 11.6 kN; 11.8 kN; 12.0 kN—for tablets with chitosan. Making tablets with comparative disintegrants, higher compression force was necessary to form tablets: 15.2 kN; 15.4 kN; 15.6 kN; 15.8 kN; 16 kN; 16.2 kN; 16.4 kN (for croscarmellose sodium and sodium starch glycolate). A single punch tableting machine CPR-6 (Dott.Bonapace &Co, Cusano Milanino, Italy) was used for tablet manufacturing.

### 2.3. Physicochemical Characterization of Granules

#### 2.3.1. Determination of Densities

For density test apparatus SOTAX TD-1 (Sotax Gmbh, Lörrach, Germany) was used. A sample was placed in a 50 mL measuring cylinder and the bulk volume taken. The bulk density (BD) and tapped density (TD) were calculated as the ratio of mass to the corresponding volume. The Carr’s index (CI) and Hausner’s ratio (HR) were also calculated using Equations (1) and (2) [28,29]:

(1) $$\mathrm{CI}\;=\frac{\mathrm{TD}\;-\;\mathrm{BD}}{\mathrm{TD}}\times 100$$

(2) $$\mathrm{HR}\;=\frac{\mathrm{TD}}{\mathrm{BD}}$$

#### 2.3.2. Tablet Evaluation

Weight variation test was done by weighing 20 tablets individually, calculating the average weight and comparing the individual tablet weight to the average weight.

Drug content: For this test, 10 tablets were weighed and crushed into powder. Ten milliliters of powder were mixed with 10 mL of methanol and placed in ultrasound-assisted bath for 15 min. After this the solution was filtered through 0.22 μm membrane filter. Quantitative analysis of meloxicam was performed using Waters 2695 chromatography system (Waters, MI, USA) equipped with Waters 996 PDA detector of 350 nm absorbance [28].

#### 2.3.3. Compact Density

The diameter and thickness of 10 tablets per batch were determined using Sotax HT1 (Sotax Gmbh, Lörrach, Germany). The masses were determined using KERN EMB 200-3 analytical balance. The compact density, CD, was calculated using Equation (3) [21,22]:

(3) $$\mathrm{CD}\;=\frac{m}{\pi r2t\;}\;$$

where m = mass, r = radius and t = thickness of tablet.

#### 2.3.4. Crushing Strength

The crushing strength of 10 tablets from each batch was determined using Sotax HT1 hardness tester (Sotax Gmbh). The load applied to cause crushing was recorded and the mean crushing strength was calculated [21].

#### 2.3.5. Friability

Twenty tablets were dedusted, weighed together, and then subjected to friability test using Sotax FT2 friabilator (Sotax Gmbh) operated at 25 ± 1 rpm for 5 min. The tablets were dedusted properly again and then reweighed collectively. The difference in weight was determined and the friability (F) value was calculated as a ratio of change in weight to original weight expressed in percentage using Equation (4) [13,28].

(4) $$F\;=\frac{\left(m_{1}-{\;m}_{2}\right)}{m_{1}}\times 100$$

where:

F—friability (proc);

m_1_—initial tablet weight (g);

m_2_—tablet weight after friability test (g).

#### 2.3.6. Disintegration Time

Six tablets from each batch were subjected to disintegration test in a freshly prepared distilled water and phosphate buffer (pH 7.2) at 37 ± 0.5 °C using disintegration test apparatus Sotax DT-2 (Sotax Gmbh) with 900 mL of distilled water without disk. The disintegration times were taken and the mean disintegration time was calculated [14,21,22].

Wetting time: A piece of tissue paper of 10 cm diameter was placed in a 10 cm diameter Petri dish containing 10 mL of water. A tablet was put on the paper. A time required for water to reach upper surface of the tablet was noted as the wetting time.

Water absorption ratio test was done following the same procedure as for the wetting time. A tablet was weighed and put on the paper in a Petri dish. When water reached the top surface of the tablet and it was completely wet, the tablet was weighed again. Water absorption ratio (R) was calculated according to Equation (5):

(5) $$R\;=\frac{V_{b}-{\;V}_{a}}{V_{a}}\times 100\;$$

where:

V_a_—tablet weight before water absorption;

V_b_—tablet weight after water absorption.

In vitro drug release: In vitro meloxicam release of from orally disintegrating tablets was determined using USP Dissolution Apparatus (Paddle type, model, Sotax AT7, Sotax Gmbh) [28,29,30]. A volume of 700 mL of phosphate buffer (pH 7.2), a dissolution medium at 37 ± 0.5 °C, was added to the dishes of the apparatus. Blade rotation speed 50 rpm. Samples were taken after 2; 4; 6; 8; and 10 min of testing. Sample volume was 5 mL. The volume of the taken samples was replaced by fresh dissolution medium. The samples were filtrated before chromatographic analysis. The analysis of samples was performed using high performance liquid chromatography (HPLC) method. A Waters 2695 chromatography system (Waters, Milford, MA, USA) equipped with Waters 996 PDA detector of absorbance at 350 nm was used.

In the oral cavity, the pH is maintained near neutrality (6.7–7.3) by saliva [31]. The dissolution test of the tablets was performed in a phosphate buffer solution with a pH of 7.2. The used medium did not affect wetting time, solution absorption, and disintegration time of meloxicam tablets.

Stability testing: Accelerated stability testing was performed for 6 months as per ICH guidelines [32]. The optimized formulations were kept at 40 ± 2 °C and 75 ± 5% RH. Physical changes, tablet hardness, and disintegration time changes were assessed every 3 months.

### 2.4. HPLC Analysis

For the quantitative analysis of meloxicam, a Waters 2695 chromatograph with a Waters 996 photodiode array detector (Waters, Milford, MA, USA) was used. An ACE C18 column (250 × 4.6 mm, sorbent particle size 5 μm) was used (Advanced Chromatography Technologies, Aberdeen, Scotland). Gradient elution was used. Mobile phase: 0.05% trifluoroacetic acid and acetonitrile, flow rate 1.0 mL/min, analysis time 24 min. The injection volume of the test solution was 10 μL. The wavelength of light used by the UV detector λ = 350 nm.

### 2.5. Statistical Analysis

Statistical analysis was performed by one-way analysis of variance (ANOVA) followed by Tukey’s multiple comparison tests; data mean and standard deviation were calculated using the software SPSS Statistics 21.0 (IBM Corporation, New York, NY, USA). A value of *p* < 0.05 was taken as the level of significance.

## 3. Results and Discussion

### 3.1. Physical Characterization of Tablets

The control F1 formulation was prepared without disintegrant. F2 formulation was prepared with chitosan, F3 with croscarmellose sodium, and F4 with sodium starch glycolate. Sorbitol was used as a diluent because of its suitable physical, mechanical properties. Sorbitol is chemically inert and is compatible with most excipients. It also has 60% of the sweetening activity of sucrose and is used as a sugar replacement in diabetes. This filler perfectly masked the unpleasant taste of meloxicam, leaving a good feeling in the mouth after taking the tablet. In the study, the data of the F2 formulation were compared with the data of the control formulation F1 without disintegrant. F3 and F4 formulations with other disintegrants—croscarmellose sodium and sodium starch glycolate—were used to compare final results in order to prove, that chitosan works as good as commonly used disintegrants. The physical properties of prepared granules are shown in Table 2.

The bulk density of the granules was not significantly different; meanwhile, tapped density was highest for the F1 formulation. This result helped in calculating the Carr’s index and Hausner’s ratio of the granules to evaluate the flowability of granules. The Carr’s index of the F1 formulation was 19.31%, F3 formulation—16.05% and F4—15.78% and it was rated as a medium flow while the Carr‘s index of the F2 formulation produced with chitosan was 10.26% and it was rated as a good flow. The same results were confirmed by the Hausner’s ratio, which had values of 1.221, 1.104, 1.149, and 1.178, respectively. This indicated that the addition of disintegrant improved the physical properties of granules. The best flowability was observed in formulation F2—containing 7% of chitosan (Carr‘s index = 10.26%, Hausner‘s ration 1104).

### 3.2. Post Formulation Studies

The prepared tablets were evaluated for weight variation, drug content, compact density, hardness, friability, disintegration time, wetting time, water absorption ratio, and in vitro dissolution. The work of Aucamp and Campus [33] showed that the presence of chitosan in tablet formulation caused a decrease in tablet strength. In order to combine proper mechanical strength and rapid disintegration and dissolution of the tablets, different compression forces (10.6 kN; 10.8 kN; 11.0 kN; 11.2 kN; 11.4 kN; 11.6 kN; 11.8 kN; 12.0 kN) were used to compress the tablets with chitosan. Compression forces, needed to form tablets with croscarmellose sodium and sodium starch glycolate were 15.2 kN; 15.4 kN; 15.6 kN; 15.8 kN; 16 kN; 16.2 kN;16.4 kN (lower compression force was not enough to form tablets with these disintegrants).

The weight of tablets (shown in Table 3) varied between 197.1 ± 0.012 mg to 202.2 ± 0.006 mg. The variation in weight was within the range of ± 7.5% complying with European Pharmacopoeia specification. The drug content varied between 94.40% ± 0.74 to 103.7% ± 0.35 for formulations within acceptable limits. 

The physical properties of tablets are shown in Table 4. 

The compact density of the F2 tablets ranged from 0.89 ± 0.01 (g/cm^3^) to 1.03 ± 0.01 (g/cm^3^) and was lower compared to the F1, F3 and F4 formulation tablets. This indicates that tablets of formulation with chitosan of all compressions were less compact compared to the same compression tablets of formulation without chitosan and higher compression tablets of formulations with croscarmellose sodium and sodium starch glycolate.

The crushing strength of all F1 formulation tablets was statistically significantly (*p* < 0.05) higher than the crushing strength of F2, F3, and F4 formulations tablets. The crushing strength of the F1 formulation tablets ranged from 3.20 to 13.00 kg/cm^2^, F2 formulation tablets ranged from 0.85 to 10.66 kg/cm^2^, F3 0.9 to 3.87 kg/cm^2,^ F4 1.2 to 3.67 kg/cm^2^. Applying a compression force of 10.6 kN to the F2 formulation tablets yielded mechanically unstable tablets, but the F1 formulation tablets made with the same compression force exhibited adequate crushing strength. Tablets made without the disintegrant were mechanically stronger, which means that the disintegrant reduced the crushing strength of the tablets. It is important to mention that tablets made with croscarmellose sodium (0.9 to 3.87 kg/cm^2^) or sodium starch glycolate (1.2 to 3.67 kg/cm^2^) had significantly lower crushing strength, even using higher compression force for tablet compression than the tablets made with lower compression force using chitosan as a disintegrant (0.85–10.66 kg/cm^2^).

Friability value of less than 1% is required for a tablet to pass the friability test. The European Pharmacopoeia requirements of the friability test were met by the formulations F1 and F2 tablets that were made using a compression force of at least 10.8 kN. There was no significant difference, but the friability of all F2 formulation tablets was higher compared to F1 formulation without chitosan tablets. The European Pharmacopoeia requirements for the friability test were met by formulation F3 with 15.8 kN compression force or higher, also formulation F4 with 16.0 kN or higher compression force. Studies have shown that the polymer chitosan tends to increase the tablet friability and decrease the mechanical strength. Therefore, when producing an orally disintegrating tablet it is important to select the correct compression force in the tablet press. Many researchers have pointed out that common problems encountered in the manufacture of fast-disintegrating tablets are associated with the low mechanical resistance of the tablets, high abrasion, and low crushing strength [1,9,34,35].

Olorunsola et al. have indicated that chitosan works by capillary action or wicking. Rapid disintegration of tablets is achieved even at low concentrations of chitosan, which proves the effectiveness of chitosan as a disintegrant. However, the authors also point out that the mechanical strength of the tablets depends on the chitosan concentration, at higher chitosan concentrations the mechanical strength of the tablets decreases [14]. Therefore, in production of orally disintegrating tablets with chitosan, due to the higher water inflow, it was necessary to form a matrix of tablets of lower compactness but with adequate mechanical strength. For this purpose, granules were produced and different compression forces were used in the tablet press. Wetting time, water absorption, and disintegration time were evaluated to determine whether the polymer chitosan and the compression force in the tablet press affected water entry into the tablet and disintegration. The wetting time, water absorption, and disintegration time of the meloxicam tablets are shown in Table 5.

There was a statistically significant difference in the wetting time and absorption between the tablets of formulations F1 and F2. F1 tablets had a longer wetting time and a lower water absorption than F2 formulation tablets of all applied compression forces. The highest water absorption and the shortest wetting time were found in F2 tablets of the lowest compression (10.6 kN): water absorption 40.94 ± 1.41% and wetting time 11.65 ± 0.87 s, but these tablets were mechanically unstable. Nagar M and others [36] have also shown in their studies that wetting time and water absorption are in direct correlation with the hardness of the tablet, i.e., wetting time increased with the increase in hardness of the tablet.

Similar water absorption was observed between different disintegrants: 40.94–10.74% chitosan; 40.76–9.81% croscarmellose sodium; 55.35–22.94% sodium starch glycolate, but the wetting time was significantly shorter in F3 and F4 formulations (even when higher compression forces were used). Disintegration time of croscarmellose sodium or sodium starch glycolate tablets was also significantly shorter comparing with chitosan.

Tablets of suitable mechanical strength with the highest water absorption and the shortest wetting time were F2 formulation with compressive forces in the tablet press of 10.8 and 11.0 kN, F3 formulation with compressive forces in the tablet press 15.8 kN or higher, F4 formulation with compressive forces in the tablet press of 16.00 kN. A statistically significant difference in disintegration time was observed between F1 and F2 formulations using different compression forces. None of the formulation F1 tablets met the requirements for fast disintegrating tablets of the disintegration time to be up to 1 min. Only those of formulation F2 tablets that were produced using 10.6, 10.8, and 11.0 kN compression forces met the requirements of disintegration test. The study proved that a polymer chitosan and the lower compression force in the tablet press shortened the wetting time of the tablets, increased the water absorption, and shortened the disintegration time of the tablets.

**Figure 2:**
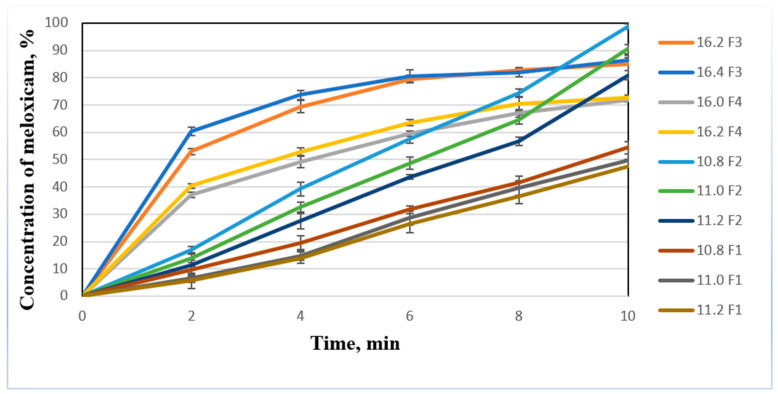
Dissolution profile of meloxicam tablets (*n* = 3, data presented as mean ± SEM. F1 = formulation without disintegrant, F2 = formulation containing 7% chitosan, F3 = formulation containing 7% croscarmellose sodium, F4 = formulation containing 7% sodium starch glycolate).

All of the F3 formulation tablets and F4 formulation tablets with compression force 16.2 or lower met the requirements for the disintegration time (tablets should disintegrate within 1 min), but only F3 formulation tablets with 15.8 kN or higher and F4 formulation tablets with 16.0 kN or higer compression force had appropriate mechanical strength. Based on previous research results, dissolution studies were performed with F1 and F2 tablet formulations that were prepared using 10.8, 11.0, and 11.2 kN compression forces in the tablet press. F3 formulation—16.2 and 16.4 kN, F4 formulation—16.0 and 16.2 kN. The results of the study are shown in the Figure 2.

The highest amount of meloxicam was released from the F2 formulation tablets that were prepared using 10.8 kN compression force. After 10 min of this test, the cumulative percent of meloxicam released from this series of tablets was 98.68 ± 0.63%. As the compression force in the tablet press increased, the release of meloxicam from the F2 formulation tablets decreased. From all F1 tablets meloxicam was released statistically significantly less compared to F2, F3, or F4 tablets. These results suggested that chitosan and lower compression force of the tablet press made the dissolution faster. Dissolution profile of croscarmellose sodium or sodium starch glycolate is similar: rapid release of meloxicam in first 4 min and then slowly reaching peak: 72.55% 16.2 F4 formulation for sodium starch glycolate and 86.51% 16.4 F3 for croscarmellose sodium. There is no statistically significant difference between different compression forces for these disintegrants. Chitosan had slower release of meloxicam in the first minutes, but the peak concentration was significantly higher (98.68%—10.8 F2). Thus, the results of our study demonstrated the similar efficacy of medium molecular weight chitosan as a disintegrant compared to the synthetic disintegrants [22].

### 3.3. Stability Testing

The F2 formulation tablets that were made using compression force of 10.8 kN; 11.0 kN; and 11.2 kN, F3 formulation tablets with 16.2 kN; 16.4 kN compression force also F4 formulation tablets with 16.0 kN and 16.2 kN compression were subjected to stability test according to ICH guidelines at 40 ± 2°/75% RH ± 5% condition in stability chamber CLIMACELL (Medcenter Einrichtungen GmbH, Munich, Germany) for 6 months. Tablets were evaluated for physical appearance, hardness (kg/cm^2^), drug content, dissolution, and disintegration time (s). The results are shown in Table 6. Tablets have not shown any significant change during storage. It was concluded that the formulation F2 tablets made with compression force 10.8 kN; 11.0 kN; 11.2 kN have a good stability during their shelf life.

## 4. Conclusions

The results showed that the polymer chitosan improves the physical properties of the meloxicam granules from which the meloxicam tablets were made but reduces the mechanical strength of the tablets by increasing the tablet wear and reducing the tablet crushing strength. It has been found that a properly selected compression force allows the production of high-quality fast disintegrating tablets. Comparing results with commonly used disintegrants supports the idea that tablets with different disintegrants often need compression force adjustments, that allow to obtain European Pharmacopeia specifications satisfying orally disintegrating tablets. Medium molecular weight chitosan can be used as a super disintegrant in the manufacture of fast disintegrating tablets of meloxicam by wet granulation method when an appropriate compression force is applied in the tablet press. The prepared fast disintegrating tablets of meloxicam with chitosan showed good mechanical and disintegration properties and a good dissolution rate when 10.8 kN and 11.0 kN compression force was used in a tablet press.

## Figures and Tables

**Table 1:** Composition of fast disintegrating tablets of Meloxicam prepared by wet granulation method.

No.	Ingredients (mg)	Formulations			
		F1	F2	F3	F4
1.	Meloxicam	7.5	7.5	7.5	7.5
2.	Chitosan	-	14	-	-
	Intragranular	-	7	-	-
	Extragranular	-	7	-	-
3.	Croscarmellose sodium	-	-	14	-
	Intragranular	-	-	7	-
	Extragranular	-	-	7	-
4.	Sodium starch glycolate	-	-	-	14
	Intragranular	-	-	-	7
	Extragranular	-	-	-	7
5.	Microcrystalline cellulose	27.5	27.5	27.5	27.5
6.	Magnesium stearate	2	2	2	2
7.	Sorbitol	134	120	120	120
	Intragranular	122.5	115.5	115.5	115.5
	Extragranular	11.5	4.5	4.5	4.5
8.	Mannitol	29	29	29	29
Total weight:		200	200	200	200

**Table 2:** The physical properties of meloxicam granules of formulations F1, F2, F3 and F4.

Properties	Formulations			
	F1	F2	F3	F4
Tapped density (g/cm^3^)	0.708 ± 0.037	0.571 ± 0.043	0.75 ± 0.074	0.833 ± 0.065
Bulk density (g/cm^3^)	0.567 ± 0.033	0.513 ± 0.029	0.601 ± 0.039	0.667 ± 0.025
Carr‘s index (%)	19.31 ± 0.89	10.26 ± 0.76	16.05 ± 1.02	15.78 ± 0.94
Hausner’s ratio	1.221 ± 0.367	1.104 ± 0.413	1.149 ± 0.281	1.178 ± 0.547

*n* = 3, data presented as mean ± SEM. F1 = formulation without disintegrant, F2 = formulation containing 7% chitosan, F3 = formulation with croscarmellose sodium, F4 = formulation with sodium starch glycolate.

**Table 3:** Weight variation and drug content of meloxicam tablets.

Compression Force, kN	Average Weight (mg) (*n* = 20)		Drug Content (%) (*n* = 10)	
	F1	F2	F1	F2
10.6	0.201 ± 0.006	0.198 ± 0.007	95.5 ± 0.33	99.3 ± 0.74
10.8	0.202 ± 0.007	0.200 ± 0.007	97.1 ± 0.18	98.7 ± 0.31
11.0	0.199 ± 0.004	0.200 ± 0.006	99.7 ± 0.55	101.4 ± 0.77
11.2	0.202 ± 0.007	0.202 ± 0.005	101.3 ± 0.17	99.7 ± 0.46
11.4	0.201 ± 0.008	0.201 ± 0.007	94.4 ± 0.74	97.6 ± 0.71
11.6	0.199 ± 0.009	0.202 ± 0.006	103.7 ± 0.35	99.1 ± 0.36
11.8	0.201 ± 0.007	0.200 ± 0.008	98.9 ± 0.73	98.7 ± 0.39
12.0	0.197 ± 0.012	0.199 ± 0.015	102.6 ± 0.69	97.1 ± 0.46
	F3	F4	F3	F4
15.2	0.201 ± 0.0017	0.200 ± 0.0024	99.2 ± 0.47	98.1 ± 0.42
15.4	0.200 ± 0.0014	0.201 ± 0.0016	96.5 ± 0.15	102.0 ± 0.88
15.6	0.200 ± 0.0018	0.201 ± 0.0018	98.8 ± 0.68	98.6 ± 0.62
15.8	0.200 ± 0.0018	0.201 ± 0.0018	98.3 ± 0.71	99.7 ± 0.31
16.0	0.200 ± 0.0016	0.201 ± 0.0009	101.6 ± 0.39	97.5 ± 0.12
16.2	0.201 ± 0.0015	0.201 ± 0.0018	97.9 ± 0.77	99.1 ± 0.42
16.4	0.200 ± 0.0017	0.200 ± 0.0014	99.4 ± 0.74	101.5 ± 0.81

*n* = 3, data presented as mean ± SEM. F1 = formulation without disintegrant, F2 = formulation containing 7% chitosan, F3 = formulation containing 7% croscarmellose sodium, F4 = formulation containing 7% sodium starch glycolate.

**Table 4:** The physical properties of meloxicam tablets of formulations F1, F2, F3, and F4.

Compression Force, kN	Compact Density (g/cm^3^)		Crushing Strength (kg/cm^2^)		Friability (%) ±SD (*n* = 20)	
	F1	F2	F1	F2	F1	F2
10.6	0.93 ± 0.01	0.89 ± 0.01	3.20 ± 0.14 *	0.85 ± 0.84	1.70 ± 0.03 *	2.00 ± 0.05
10.8	0.94 ± 0.01	0.92 ± 0.01	4.91 ± 0.22 *	3.00 ± 4.41	0.48 ± 0.05 *	0.94 ± 0.03
11.0	0.96 ± 0.01	0.94 ± 0.00	6.00 ± 0.16 *	3.63 ± 3.14	0.47 ± 0.05	0.50 ± 0.05
11.2	0.99 ± 0.00	0.96 ± 0.00	7.35 ± 0.15 *	4.17 ± 5.68	0.46 ± 0.03	0.48 ± 0.05
11.4	1.00 ± 0.01	0.97 ± 0.01	9.38 ± 0.21 *	4.33 ± 6.77	0.45 ± 0.04	0.47 ± 0.03
11.6	1.01 ± 0.00	1.00 ± 0.01	10.90 ± 0.16 *	7.61 ± 3.14	0.43 ± 0.07	0.44 ± 0.01
11.8	1.02 ± 0.01	1.01 ± 0.01	12.31 ± 0.14 *	8.43± 5.10	0.42 ± 0.05	0.43 ± 0.03
12.0	1.04 ± 0.01	1.03 ± 0.01	13.00 ± 0.15 *	10.66 ± 6.11	0.41 ± 0.05	0.42 ± 0.05
	F3	F4	F3	F4	F3	F4
15.2	1.24 ± 0.01	1.20 ± 0.02	0.9 ± 1.93 ^+^	-	6.79 ± 1.17 ^+^	49.8 ± 3.7
15.4	1.45 ± 0.02	1.44 ± 0.02	1.25 ± 1.85 ^+^	1.20 ± 1.6	6.06 ± 0.67	3.49 ± 0.18
15.6	1.50 ± 0.02	1.47 ± 0.01	1.60 ± 2.02 ^+^	1.52 ± 1.26	1.69 ± 0.08 ^+^	3.29 ± 0.17
15.8	1.55 ± 0.02	1.53 ± 0.01	2.50 ± 2.25 ^+^	2.04 ± 1.92	0.87 ± 0.03	1.22 ± 0.067
16.0	1.61 ± 0.01	1.58 ± 0.01	2.90 ± 0.76 ^+^	2.59 ± 1.83	0.67 ± 0.041	0.98 ± 0.066
16.2	1.62 ± 0.01	1.60 ± 0.02	3.48 ± 2.02 ^+^	3.03 ± 2.5	0.52 ± 0.12	0.78 ± 0.05
16.4	1.64 ± 0.02	1.63 ± 0.02	3.87 ± 1.22 ^+^	3.67 ± 2.06	0.32 ± 0.07	0.48 ± 0.037

*n* = 3, data presented as mean ± SEM. F1 = formulation without disintegrant, F2 = formulation containing 7% chitosan; F3 = formulation containing 7% croscarmellose sodium, F4 = formulation containing 7% sodium starch glycolate. * *p* < 0.05 vs. F2, ^+^ *p* < 0.05 vs. F4.

**Table 5:** Wetting time, water absorption, and disintegration time of meloxicam tablets of formulations F1, F2, F3, and F4.

Compression Force, kN	Wetting Time (s)		Water Absorption, %		Disintegration Time (s)	
	F1	F2	F1	F2	F1	F2
10.6	789.31 ± 4.1	11.65 ± 0.87	7.68 ± 0.98	40.94 ± 1.41	80.86 ± 0.58 *	18.65 ± 1.2
10.8	971.98 ± 1.6 *	16.95 ± 0.88	7.53 ± 0.74	36.9 ± 0.98	119.34 ± 0.19 *	40.01 ± 1.12
11.0	1057.3 ± 3.6 *	23.38 ± 2.13	6.85 ± 0.66	32.81 ± 1.13	125.72 ± 1.88 *	59.65 ±5.75
11.2	1574 ± 3.2 *	68.13 ± 2.1	6.43 ± 0.48	22.43 ± 1.15	206.97 ± 0.45 *	106.59 ± 2.61
11.4	1698.35 ± 3.7 *	92.65 ± 1.2	6.28 ± 0.78	17.37 ± 0.74	219.44 ± 2.39 *	140.04 ±7.69
11.6	1985.78 ± 3.1 *	144.64 ± 0.71	6.01 ± 0.33	12.66 ± 0.56	260.1 ± 4.95 *	235.37 ± 2.24
11.8	2338.4 ± 2.5 *	214.8 ± 2.16	5.67 ± 0.81	10.76 ± 0.58	279.18 ± 2.8 *	296.56 ±3.61
12.0	2541.8 ± 3.1 *	256.55 ± 1.88	5.14 ± 0.45	10.74 ± 0.74	394.32 ± 5.36 *	353.34 ± 5.14
	F3	F4	F3	F4	F3	F4
15.2	11.68 ± 0.81 ^+^	6.54 ± 0.63	40.76 ± 1.45 ^+^	55.35 ± 1.53	12.82 ± 0.83 ^+^	16.68 ± 0.618
15.4	16.18 ± 0.79	12.57 ± 1.47	29.43 ± 1.35	40.5 ± 1.47	17.86 ± 0.66	18.05 ± 0.96
15.6	18.9 ± 1.11	15.75 ± 0.76	31.65 ± 1.28	34.84 ± 1.11	18.96 ± 1.65	22.69 ± 1.16
15.8	27.37 ± 2.09	18.71 ± 1.45	24.36 ± 1.43	35.96 ± 0.86	21.28 ± 0.83	27.16 ± 1.41
16.00	29.56 ± 1.71	20.71 ± 1.26	23.33 ± 1.52	34.15 ± 1.62	22.16 ± 1.91	35.73 ± 0.83
16.2	51.64 ± 1.56 ^+^	21.25 ± 2.15	17.4 ± 1.45 ^+^	30.99 ± 1.81	41.74 ± 0.8 ^+^	54.49 ± 1.24
16.4	58.78 ± 0.73 ^+^	42.58 ± 3.29	9.81 ±1.62 ^+^	22.94 ± 1.69	45.15 ± 2.53 ^+^	60.82 ± 1.52

*n* = 3, data presented as mean ± SEM. F1 = formulation without disintegrant, F2 = formulation containing 7% chitosan, F3 = formulation containing 7% croscarmellose sodium, F4 = formulation containing 7% sodium starch glycolate.; * *p* < 0.05 vs. F2, ^+^ *p* < 0.05 vs. F4.

**Table 6:** Stability data of F2 formulation (compression force 10.8 kN; 11.0 kN; 11.2 kN) F3 formulation (compression force 16.2 kN; 16.4 kN), F4 formulation (compression force 16.0 kN, 16.2 kN). at 40 °C/75% RH.

Formulations	Evaluation Parameters	Duration in Months		
		0	3	6
F2-10.8 kN	Physical changes	No changes	No changes	No changes
	Hardness (kg/cm^2^)	3.00 ± 4.41	2.97 ± 2.93	2.97 ± 2.75
	Disintegration time (s)	40.01 ± 1.12	39.78 ± 0.93	39.69 ± 0.75
	Drug content (%)	98.70 ± 0.31	98.30 ± 0.28	98.40 ± 0.30
	Dissolution (%)	98.68 ± 0.63	98.45 ± 0.53	98.60 ± 0.68
F2-11.0 kN	Physical changes	No changes	No changes	No changes
	Hardness (kg/cm^2^)	3.63 ± 3.14	3.62 ± 2.71	3.62 ± 3.03
	Disintegration time (s)	59.65 ± 5.75	59.62 ± 6.71	59.54 ± 6.03
	Drug content (%)	101.40 ± 0.77	101.00 ± 0.65	101.2 ± 0.67
	Dissolution (%)	90.54 ± 1.71	90.37 ± 1.68	90.40 ± 1.65
F2-11.2 kN	Physical changes	No changes	No changes	No changes
	Hardness (kg/cm^2^)	4.17 ± 5.68	4.15 ± 1.99	4.14 ± 1.26
	Disintegration time (s)	106.59 ± 2.61	106.53 ± 1.79	106.46 ± 1.16
	Drug content (%)	99.70 ± 0.46	99.50 ± 0.35	99.30 ± 0.42
	Dissolution (%)	80.84 ± 1.67	80.72 ± 1.58	80.69 ± 1.72
F3-16.2 kN	Physical changes	No changes	No changes	No changes
	Hardness (kg/cm^2^)	3.48 ± 2.02	3.47 ± 2.90	3.42 ± 2.75
	Disintegration time (s)	41.74 ± 0.80	40.76 ± 0.90	39.91 ± 0.65
	Drug content (%)	97.90 ± 0.74	97.30 ± 0.88	98.0 ± 0.79
	Dissolution (%)	84.97 ± 2.41	84.17 ± 2.73	83.95 ± 2.54
F3-16.4 kN	Physical changes	No changes	No changes	No changes
	Hardness (kg/cm^2^)	3.87 ± 1.22	3.79 ± 1.71	3.82 ± 1.53
	Disintegration time (s)	45.15 ± 5.65	46.52 ± 6.70	46.74 ±5.03
	Drug content (%)	99.40 ± 0.42	98.60 ± 0.98	98.2 ± 0.77
	Dissolution (%)	86.51 ± 1.74	85.81 ± 1.96	85.04 ± 2.05
F4-16.0 kN	Physical changes	No changes	No changes	No changes
	Hardness (kg/cm^2^)	2.59 ± 1.83	2.63 ± 1.90	2.60 ± 1.75
	Disintegration time (s)	35.73 ± 0.83	36.70 ± 0.93	36.59 ± 0.75
	Drug content (%)	97.50 ± 0.39	98.20 ± 0.55	98.01 ± 0.27
	Dissolution (%)	71.97 ± 0.79	71.31 ± 1.46	70.07 ± 1.82
F4-16.2 kN	Physical changes	No changes	No changes	No changes
	Hardness (kg/cm^2^)	3.03 ± 2.5	3.22 ± 2.70	3.16 ± 2.13
	Disintegration time (s)	54.49 ± 1.24	54.92 ± 1.71	55.24 ±1.03
	Drug content (%)	99.1 ± 0.81	99.80 ± 0.56	98.6 ± 0.73
	Dissolution (%)	72.55 ± 1.15	71.81 ± 1.71	71.22 ± 2.66

*n* = 3, data presented as mean ± SEM.

## Data Availability

The data presented in this study is available on request from the authors.
